# Whole Brain Radiation Therapy Plus Stereotactic Radiosurgery in the Treatment of Brain Metastases Leading to Improved Survival in Patients With Favorable Prognostic Factors

**DOI:** 10.3389/fonc.2019.00205

**Published:** 2019-03-29

**Authors:** Muhammad Khan, Jie Lin, Guixiang Liao, Yunhong Tian, Yingying Liang, Rong Li, Mengzhong Liu, Yawei Yuan

**Affiliations:** ^1^Department of Radiation Oncology, Affiliated Cancer Hospital & Institute of Guangzhou Medical University, Guangzhou, China; ^2^Department of Oncology, First Affiliated Hospital of Anhui Medical University, Hefei, China; ^3^Department of Radiation Oncology, Sun Yat-sen University Cancer Center, Sun Yat-sen Medical University, Guangzhou, China

**Keywords:** whole brain radiotherapy (WBRT), stereotactic radiosurgery (SRS), overall survival (OS), prognostic factors (PF), brain metastases (BM)

## Abstract

**Background:** Significantly better local control is achieved with combination of whole brain radiotherapy and stereotactic radiosurgery in the treatment of multiple brain metastases. However, no survival benefit was reported from this advantage in local control.

**Objective:** The objective of this study was to review the available evidence whether better local control achieved with whole brain radiotherapy plus stereotactic radiosurgery leads to any benefit in survival in patients with favorable prognostic factors.

**Methods and Materials:** Electronic databases (PubMed, MEDLINE, and Cochrane Library) were searched until Oct 2018 to identify studies published in English that compared efficacy of whole brain radiotherapy plus stereotactic radiosurgery vs. whole brain radiotherapy alone or stereotactic radiosurgery alone in patients with brain metastases stratified on prognostic indices (Recursive Partitioning Analysis and Diagnosis-Specific Graded Prognostic Assessment). Primary outcome was survival.

**Results:** Five studies (*n* = 2728) were identified, 3 secondary analyses of the previously published RCTs and 2 retrospective studies, meeting the inclusion criteria. whole brain radiotherapy plus stereotactic radiosurgery showed improved survival in brain metastatic cancer patients with better prognostic factors particularly when compared to whole brain radiotherapy only. Its survival advantage over stereotactic radiosurgery only was limited to non-small cell lung cancer primary tumor histology.

**Conclusions:** Whole brain radiotherapy in combination with stereotactic radiosurgery may improve survival and could be recommended selectively in patients with favorable prognostic factors particularly in comparison to whole brain radiotherapy only.

## Rationale

Brain metastases are associated with poor prognosis (1). Traditionally surgery followed by whole brain radiotherapy has been the mainstay of treatment for single brain metastasis and limited systemic disease. Stereotactic radiosurgery could also be used where surgery is inadvisable (2). Stereotactic radiosurgery plus whole brain radiotherapy is usually preferred and recommended for up-to 3 brain metastases. Brain metastases more than three have generally been treated with whole brain radiotherapy alone (3). Combination of stereotactic radiosurgery and whole brain radiotherapy have yielded better local and distant control in 1 to 4 brain metastases with no survival advantage in comparison to each treatment alone (WBRT or SRS alone) (4). Some studies revealed survival advantage associated with aggressive treatment in these patients. Since no comparative studies were available to show if this advantage was in fact due to aggressive treatment or could have also been due to better selection of patients (5). This has led to undertaking of studies in order to identify prognostic factors associated with survival advantage in these patients. Multiple prognostic indices were developed including Radiation Therapy Oncology Group Recursive Partitioning Analysis (RTOG RPA) (6), the Rotterdam Score (7), the Scoring Index for Radiosurgery (SIR) (8), the Basic Score for Brain Metastases (BSBM) (9), the Golden Grading System (GGS) (10), 2 Rades classification (RADES) (11, 12), Graded Prognostic Assessment (GPA) (13), Diagnosis-Specific Graded Prognostic Assessment (DS-GPA) (14) and a monogram tool (15). These indices had included more or less the same prognostic factors. Most frequent ones are performance status, age, extra-cranial metastases, primary tumor control, number of brain metastases and primary tumor site and histology. The rare ones mostly included in one of the indices are volume of largest brain metastasis (SIR), time from cancer diagnosis to brain radiation (Rades) and response to steroids (Rotterdam).

Radiation Therapy Oncology Group (RTOG) recursive partitioning analysis (RPA) analyzed 1,276 patients from 3 consecutive RTOG trials (6). A number of variables were analyzed for their prognostic significance on survival outcome. Class I with a median survival of 7.1 months resulted from combination of four factors including KPS ≥70, primary tumor controlled, <65 years of age and brain metastases only. The resulted class II with a median survival of 4.2 months included patients with KPS ≥70, primary tumor uncontrolled, ≥65 years of age and extra-cranial metastases. Class III with a median survival of only 2.3 months included the patients' group with KPS <70. The RPA results were later validated in a RTOG randomized trial as well as a retrospective study.

Graded Prognostic Assessment (GPA) was developed later in 2008 by Sperduto et al. involving 1960 patients from 5 phase III RTOG trials (13). Extra cranial disease status that was included RPA was excluded due to difficulty in asserting the controlled and uncontrolled status of disease while number of brain metastases was included due to its prognostic impact on survival. Other prognostic factors included were age, KPS and presence or absence of extra-cranial metastases. Each factor was assigned 0, 0.5, or 1 value. As a result four GPA prognostic groups were created for significant median survivals of 2.6 months (GPA 0–1), 3.8 months (GPA 1.5–2.5), 6.9 months (GPA 4), and 11 months (GPA 3.5–4).

A further enhanced form of GPA—diagnosis specific graded prognostic assessment (DS-GPA) was developed in a multi-institutional analysis of 4,259 patients with newly diagnosed brain metastases from eleven institutions (14). In this analysis prognostic factors were evaluated based on primary cancer. The significant factors for non-small cell lung cancer and small cell lung cancer were KPS, age, presence of extra-cranial metastases and number of brain metastases. KPS and the number of brain metastases were the significant prognostic factors for melanoma and renal cell carcinoma. For breast and GI cancers, the KPS was the only prognostic factor.

The aim of this study is to evaluate the impact of prognostic factors on the selection of treatment modality for patients with brain metastases with ultimate goal of improving patient selection process in order to achieve better outcome.

## Methods and Materials

### Eligibility Criteria

Studies published in English with no design restrictions that reported any of the following comparisons: WBRT vs. WBRT plus SRS; SRS vs. WBRT vs. WBRT plus SRS and SRS alone vs. SRS plus WBRT stratified by prognostic index (RPA, DS-GPA) for survival outcome, were eligible for inclusion. Participants with brain metastases (1–3) were eligible regardless of the primary tumor histology status. Primary outcome of interest was assessment of overall survival based on prognostic index for the treatment difference.

### Information Sources and Search Strategy

The following electronic databases were searched till Oct 2018: PubMed, MEDLINE and Cochrane Library for studies published in English language. A comprehensive research strategy was applied using various search terms including “whole brain radiotherapy” OR “whole brain radiation therapy” OR “WBRT” AND “Stereotactic Radiosurgery” OR “Radiosurgery OR SRS” AND “brain metastases” OR “brain metastasis” OR “BM” AND “Prognostic index” OR “RPA” OR “DS-GPA.” Relevant articles and abstracts were screened and reviewed, and the reference lists from those sources were searched for additional studies.

### Study Selection, Data Extraction and Quality Assessment

Eligibility assessment was performed independently by two independent reviewers in an unblinded standardized manner. In case of any disagreement, a third reviewer was consulted and issues were resolved by consensus. Full text screening was undertaken. Following data were extracted by two reviewers; Studies name, search design, number of participants, intervention comparison, prognostic index, primary tumor histology and median survival. All available data were extracted from relevant texts, tables, and figures. Quality of the RCTs was assessed by Jaded et al. method (16). A trial achieving ≥3 points was considered high quality. Newcastle-Ottawa Scale was used to assess the quality of the retrospective studies (17).

## Results

**Figure 1:**
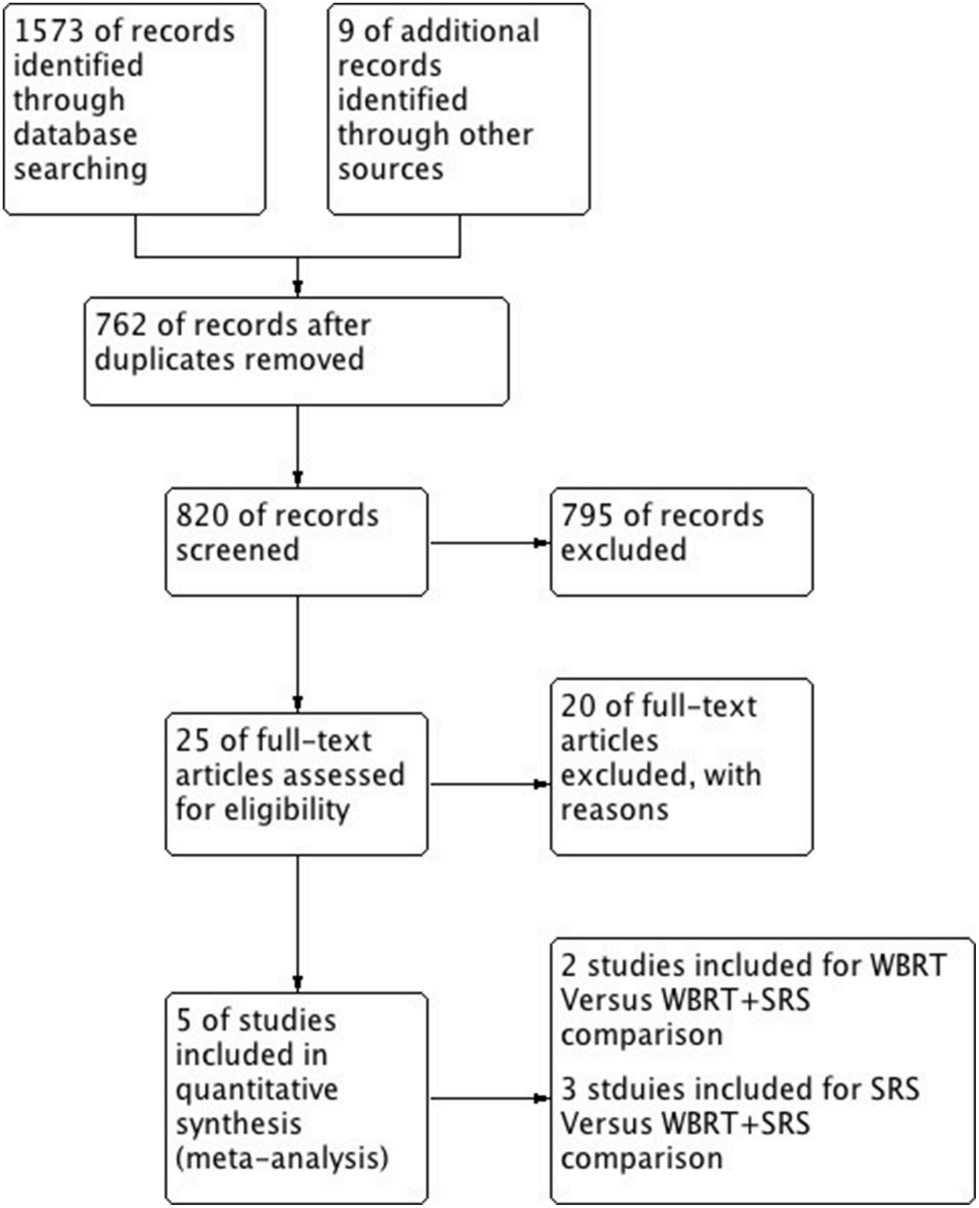
PRISMA flow diagram for the systematic review detailing the database searches, the number of abstracts screened and the full texts retrieved.

A total of 5 studies involving 2,728 participants were identified for inclusion in this systematic review (Figure 1). Table 1 has outlined general characteristics of participants and studies included. Three post-stratified graded prognostic assessment (GPA) secondary analysis of previous randomized controlled trials and two retrospective cohort studies were selected for inclusion (18–22). Diagnosis-specific graded prognostic assessment (DS-GPA) was used in the three RCTs' secondary analysis while recursive-partitioning analysis (RPA) was used for stratification in the retrospective cohort studies. Extracted data from the included studies is outlined in Table 2. WBRT plus SRS comparison to WBRT alone was based on 2 studies (1 secondary analysis of RCT & 1 retrospective study) involving 1,954 patients (18, 21). On the other hand, its comparison to SRS only was based on 3 studies (2 secondary analysis of RCT & 1 retrospective study) involving 783 patients (19, 20, 22). RCTs were rated high quality as each scored 3 points as assessed by Jaded et al. method and both retrospective studies were poor quality as each achieved 6 points (maximum 9 points) on Newcastle-Ottawa Scale (16, 17).

**Table 1:** General characteristics of the included studies.

**References**	**Study type**	**Comparison**	**Prognostic Index**	**Primary histology**
Sperduto et al. (18)	Secondary analysis (RCT)	WBRT vs. WBRT+SRS	DS-GPA	Lung, gastrointestinal, renal cancers and melanoma
Aoyama et al. (19)	Secondary analysis (RCT)	SRS vs. WBRT+SRS	DS-GPA	NSCLC
Churilla et al. (20)	Secondary analysis (RCT)	SRS vs. SRS+WBRT	DS-GPA	NSCLC
Sanghavi et al. (21)	Retrospective cohort study	WBRT vs. WBRT+SRS	RPA	Lung, breast, melanoma and others
Sneed et al. (22)	Retrospective cohort study	SRS vs. WBRT+SRS	RPA	Breast, Kidney, lung, melanoma and others

*RPA, recursive partitioning analysis; DS-GPA, Diagnosis-specific graded prognostic assessment; WBRT, whole brain radiotherapy; SRS, stereotactic radiosurgery; RCT, randomized controlled trial; NSCLC, non-small cell lung cancer*.

**Table 2:** Data extracted from the included studies.

**References**	**Study type**	**Prognostic Index**	**Comparison**				
Sperduto et al. (18)	Secondary analysis (RCT)	DS-GPA	**WBRT alone**		**WBRT+SRS**		
			*N* = 126	MST	*N* = 126	MST	*P-value*
		<3.5	104	5.4	101	5.0	0.97
		3.5–4.0	22	10.3	25	21.0	0.05^*^
Aoyama et al. (19)	Secondary analysis (RCT)	DS-GPA	**SRS alone**		**WBRT+SRS**		
			*N* = 45	MST	*N* = 43	MST	*P-value*
		0.5–2.0	19	6.5	22	4.75	0.86
		2.5–4.0	26	10.6	21	16.7	0.04^*^
Churilla et al. (20)	Secondary analysis (RCT)	DS-GPA	**SRS alone**		**SRS+WBRT**		
			*N* = 69	MST	*N* = 57	MST	*P-value*
		0.5–1.5	38	6.6	25	3.7	0.85
		2.0–4.0	31	17.9	32	11.3	0.63
Sanghavi et al. (21)	Retrospective cohort study with historical controls (Evidence class III)	RPA	**WBRT alone**		**WBRT+SRS**		
			*N* = 1200	MST	*N* = 502	MST	*P-value*
		Class I	236	7.1	112	16.1	0.05^*^
		Class II	765	4.2	356	10.3	0.05^*^
		Class III	175	2.3	34	8.7	0.05^*^
Sneed et al. (22)	Retrospective cohort study (Evidence class II)	RPA	**SRS alone**		**WBRT+SRS**		
			*N* = 268	MST	*N* = 301	MST	*P-value*
		Class I	39	14.0	64	15.2	0.98
		Class II	197	8.2	222	7.0	0.38
		Class III	29	5.3	9	5.5	0.51

RPA, recursive partitioning analysis; DS-GPA, -specific graded prognostic assessment; WBRT, brain radiotherapy; SRS, radiosurgery; RCT, controlled trial; MST , survival time; N, number of participants;

* *Statistical significance*.

### WBRT + SRS vs. WBRT

Two studies were identified for comparison of WBRT vs. WBRT plus SRS. A secondary analysis of randomized controlled trial (RTOG 9508) and a retrospective cohort study (18, 21). DS-GPA was used to classify the patients into grades for grade-based comparative analysis of survival benefit for the treatment difference. A total of 252 patients (SRS = 126, WBRT+SRS = 126) were evaluated in the secondary analysis. Primary histology included lung, gastrointestinal, renal and melanoma cancers. Two prognostic grades were achieved with DS-GPA (GPA < 3.5 & 3.5–4.0). Patients with GPA 3.5–4.0 had better OS when treated with WBRT+SRS. Median survival time was 21.0 months (2-year OS 43%) for WBRT+SRS and 10.3 months (2-year OS 21%) for WBRT alone (*p* = *0.05*). A 9.6 months survival advantage (MST; 21.0 vs. 11.4 months) was achieved when analysis was restricted to patients with single metastases only in GPA 3.5–4.0 group. Similarly, patients with GPA 3.5–4.0 and 2 or 3 metastases, a 5.2-month survival benefit (MST; 14.1 vs. 8.9 months) was achieved for treatment difference favoring WBRT plus SRS. Patients with GPA < 3.5, the 2 treatment groups revealed no difference in median survival even when no of metastases restrictions were applied.

Sanghavi et al. retrospective cohort study included 502 patients with brain metastases from lung, breast and melanoma as primary cancers were treated with WBRT followed by SRS boost. The interval between the treatments was not restricted however SRS was not given as salvage therapy but as a treatment boost. These patients were stratified into RTOG RPA classes. The three RPA classes were then compared with RTOG RPA studies (Trials 79–16, 85–28, and 89–05), which involved a total of 1,200 patients treated with WBRT only. Comparison by class revealed SRS boost resulted in better survival for each class. 16.1 months of median survival for WBRT plus SRS compared to 7.1 months for WBRT only for class I (*p* < *0.05*), 10.3 months vs. 4.2 months for class II (*p* < *0.05*) and 8.7 months vs. 2.3 months for class III (*p* < *0.05*).

### WBRT +SRS vs. SRS

Secondary analysis of JROSG 99-1 randomized trial was undertaken for stratification of patients with better DS-GPA scores in order to analyze if the combined approach yields any survival advantage. Post-stratification 88 patients by DS-GPA scores with NSCLC as primary histology was done. Patients had 1 to 4 brain metastases and overall survival was the primary end point of this study (19). Significant survival advantage (HR, 1.92; 95%CI, 1.01–3.78; *p* = *0.04*) was reported for patients receiving WBRT plus SRS with high DS-GPA score of 2.5–4.0 (total *n* = 47 patients; *n* = 26 in SRS alone & *n* = 21 in WBRT + SRS). Median survival of 16.7 (95%CI, 7.5–72.9) months with WBRT plus SRS compared to 10.6 (95%CI, 7.7–15.5) months with SRS only *(p* = *0.04)*. No advantage in survival was revealed (HR, 1.05; 95%CI, 0.55–1.99; *p* = *0.86*) for patients with low DS-GPA score (0.5–2.0) for the treatment difference.

A Secondary Analysis of the NCCTG N0574 RCT included a total of 126 NSCLC patients with a median follow up of 14.2 months (20). Baseline characteristics of the patients were well-matched between the groups (SRS vs. SRS+WBRT) with regard to DS-GPA. No survival advantage was revealed between the treatments in both DS-GPA groups (0.5–1.5 (HR, 0.95; 95%CI, 0.56–1.62; *p* = *0.85*) vs. (HR, 0.86; 95%CI, 0.47–1.59; *p* = *0.85*) >2.0–4.0). No significant survival (*p* = *0.53*) was achieved with combined approach even when analyses were restricted to favorable prognostic patients (DS-GPA ≥ 2.5).

A retrospective cohort study by Sneed et al. compared SRS alone vs. SRS plus WBRT for patients with brain metastases (22). Overall 589 patients were included in this study with 268 patients received SRS only (24% had received WBRT as salvage therapy later) and 301 had SRS + up-front WBRT. Patients were stratified into RTOG RPA classes. This study reported no survival advantage for treatment difference when stratified RPA classes were compared (14.0 vs. 15.2 months for RPA Class 1 patients, 8.2 vs. 7.0 months for Class 2, and 5.3 vs. 5.5 months for Class 3, respectively, *p* = 0.33, hazard ratio = 1.09).

## Discussion

Adding stereotactic radiosurgery to whole brain radiotherapy in the treatment of brain metastases is a much-debated topic over the past decade as to where this combination is better in comparison to either treatment solely. Combination has produced local and distant tumor control but it has not been translated into survival benefit (23–27). Literature research has revealed a number of prognostic factors affecting the survival outcome. It seemed inevitable to judge the treatments effect when both the treatment arms included patients with same prognostic classification class. Hence an attempt was made to stratify some of the previous randomized controlled trials based on new indices developed from combination of these prognostic factors (18–20).

Performance status, age and systemic tumor activity were the first three prognostic factors associated with survival in patients with brain metastases identified by Radiation Therapy Oncology Group (RTOG). Sanghavi et al. (21) carried out a retrospective cohort study comparing the WBRT with WBRT plus SRS based on this prognostic index (RPA). WBRT plus SRS arm was stratified into 1 of 3 RPA classes for comparison. A historical control of similar patients receiving WBRT only was chosen for class comparison. Comparative analysis revealed a significant survival benefit for patients receiving WBRT + SRS in each class with most prominent difference in RPA class I (9 months). Similarly, a retrospective cohort study (Evidence class II) compared survival probabilities of patients with newly diagnosed brain metastases based on data collected from 10 institutions (22). Patients were either treated with radiosurgery or radiosurgery plus whole brain radiotherapy. RPA classification was implied to analyze the survival advantage. No survival difference was revealed between the treatment arms (hazard ratio = 1.09, *p* = 0.33).

Previously it was assumed that type of primary histology had no impact on the brain metastatic lesions' behavior to treatment modality. However, survival benefit was observed with combined approach when Andrew et al. study was restricted to lung cancer only (24). From this result one could derive that a more logical comparison could be achieved when a diagnosis based prognostic criteria is applied. Secondary analysis of RTOG 9508 (18) was the first step taken in this direction by Sperduto et al. In this analysis, DS-GPA was used to stratify patients to analyze for treatment difference. A statistically significant survival was reported in patients with high GPA (3.5–4.0) regardless of the number of metastases. Median survival time for WBRT +SRS was 21 months as compared to 10.3 months with WBRT alone (*p* = 0.05). Sanghavi et al. also reported a significantly high median survival in RPA class I for patients receiving combined therapy approach. These results recommend patients with better prognosis could undertake aggressive treatment with combining both the treatment modalities in order to achieve better survival.

This review revealed survival benefit for patients with brain metastases receiving combined modalities regardless of the number of brain metastases when compared to WBRT alone if based on prognostic criteria (RPA or DS-GPA). However, a number of other studies have also reported better survival regardless of the prognostic classification (28–31). Two RCTs (28, 29) revealed significantly better survival for the combined approach as compared to WBRT only. Wang et al. (30) reported better survival (91 vs. 37 weeks, *p* < 0.00001) for patients opting to receive aggressive treatment. Hyun et al. (31) undertook a meta-analysis and reported comparatively better survival in patients receiving WBRT + SRS (10.7 vs. 6 m). A Survival advantage for patients with single brain metastasis has already been reported receiving WBRT+SRS in comparison to WBRT alone by Andrews et al. (24) and Li et al. (32) regardless of any prognostic classification. These studies have proved that patients receiving WBRT plus SRS can derive better survival benefit in comparison to WBRT only particularly if patient selection is based on prognostic classification.

On the other hand, SRS only has shown to be much more comparative than WBRT alone. Sneed et al. (33) didn't report any significant survival in patients with better prognosis. However, Aoyama et al. (19) revealed survival benefit for patients with single as well as multiple brain metastases in WBRT+SRS arm with high GPA class (2.5–4.0). Churilla et al. secondary analysis also didn't reveal any survival advantage for highly prognostic patients (>2.5) (20). it must be noted that this study had much fewer patients in highly prognostic DS-GPA group (>2.5) compared to Aoyama et al. Medical literature revealed contrast results regarding survival benefit when SRS alone was compared to WBRT + SRS. Wang et al. (30) reported significant survival advantage for patients receiving WBRT + SRS (91 vs. 67 w). One meta-analysis (31) and one retrospective (34) reported better survival however was not significant. A number of other studies either reported equal or better survival for SRS alone regardless of the prognostic classification (25–27, 34–36). Li et al. reported no survival benefit for patients with single brain metastasis receiving WBRT+SRS when compared to SRS alone (32). The RCTs comparing SRS vs. WBRT+SRS have not reported comparative analyses of patients with single brain metastasis for treatment difference (26, 27). Several studies have reported median survival comparisons for these treatments as shown in Table 3. WBRT plus SRS is shown to be significantly beneficial in some of these studies when compared to WBRT alone. However, its comparison with SRS only has been shown to be statistically insignificant.

**Table 3:** Comparison of median survival reported from different studies comparing WBRT, SRS, and WBRT+SRS.

**References**	**Study type**	**WBRT**	**SRS**	**WBRT+SRS**	***P-values***
Andrews et al. (24)	RCT	5.7		6.5	0.1356
Kondziolk et al. (23)	RCT	7.5		11	0.226
Minniti et al. (29)	RCT	7.2		10.3	0.005^*^
Lin et al. (28)	RCT	0.53 yrs.		1.46 yrs.	<0.0001^*^
Wang et al. (30)	RC	37 wks.	67 wks.	91 wks.	<0.00001^*^
Chougule et al. (35)	RCT	9	7	5	N/A
Min Kyung Hyun et al. (31)	Meta	6	7.9	10.7	N/A
Aoyama et al. (26)	RCT		8	7.5	0.42
Sneed et al. (33)	RC		11.3	11.1	NS
Noel et al. (34)	RC		7	14	NS
Brown et al. (27)	RCT		10.4	7.4	0.92
Hoffman et al. (36)	RC		13.9	14.5	NS

*WBRT, whole brain radiotherapy; SRS, stereotactic radiosurgery; RCT, randomized controlled trial; Meta, meta-analysis; yrs., years; wks., Weeks; NS, not significant; N/A, not available*.

* *Statistical significance*.

Stereotactic radiosurgery alone results in significantly high need for salvage therapy (20, 26, 27). High salvage treatment requirement could possibly have mental and economic implications for patients. This needs to be accounted for when deciding proper therapy for patients with brain metastases. Patients with better prognosis should therefore be recommended a combined approach in order to maintain a better local tumor control and distant recurrence rate (reduce the risk for future therapy need) thereby a better chance of deriving probable survival advantage. Low quality of life particularly neurocognition status remains the prime matter of concern with WBRT. Memantine is being used with WBRT in order to minimize cognitive effects of WBRT. A randomized trial (RTOG 0614) (37) of WBRT vs. WBRT plus memantine in patients with brain metastases showed better cognitive function over time in the memantine group. Hippocampal avoidance whole brain radiotherapy (HA-WBRT) has demonstrated significantly better memory preservation compared to historical controls of WBRT alone (38–40). Further strategies with promising results include RAS blockers, donepezil (Acetylcholinesterase inhibitors) and peroxisomal proliferator-activated receptor agonists (PPAR) (41).

Secondary analysis of both the RCTs mainly had patients with primary lung cancer particularly non-small cell lung carcinoma. In Sperduto et al. secondary analysis, 211 patients out of 252 had lung cancer. While Aoyama et al. secondary analysis only stratified patients with non-small lung cell carcinoma. Due to retrospective nature of the other two studies (21, 22), high selection bias could be incurred. Moreover, Sanghavi et al.'s retrospective study was conducted from 1988 to 1998 whereas historical controls (RTOG studies) it was compared to were conducted from 1976–1993. As well, the interval between SRS and WBRT was 16 weeks in some of the patients (62 (12%) of 502) leading to possible selection bias.

## Conclusions

Better local and distant tumor control achieved with WBRT followed by SRS boost resulted in significantly better survival in treatment of prognostically better placed patients with brain metastases compared to WBRT alone in particular. Preference of combined approach to SRS only in restricted to NSCLC primary histology and further assessment is needed to prove its effectiveness in other primary tumor histology. Overall this combination may represent a better choice and could be recommended to patients with 1–3 brain metastases with better prognostic class.

## Author Contributions

All authors listed have made a substantial, direct and intellectual contribution to the work, and approved it for publication.

### Conflict of Interest Statement

The authors declare that the research was conducted in the absence of any commercial or financial relationships that could be construed as a potential conflict of interest.
